# Comparison of BRCA1 Gene Expression and CA15-3 Tumor Marker Level in Different Stages of Breast Cancer

**DOI:** 10.1155/2024/3461694

**Published:** 2024-08-12

**Authors:** Negar Soltani Irdmusa, Haniyeh Bashi Zadeh Fakhar, Masoumeh Heshmati, Mohammad Esmaiel Akbari, Sara Rahimi

**Affiliations:** ^1^ Department of Cell and Molecular Sciences Faculty of Advanced Sciences and Technology Tehran Medical Science Islamic Azad University, Tehran, Iran; ^2^ Department of Laboratory Science Chalus Branch Islamic Azad University, Chalus, Iran; ^3^ Cancer Research Center Shahid Beheshti University of Medical Sciences, Tehran, Iran

## Abstract

Breast cancer (BC), a globally prevalent malignancy, shows significant variability in incidence across different geographical regions. In this study, we examined the expression of the tumor suppressor gene BRCA1 and the tumor marker CA15-3 in women diagnosed with BC, focusing on different cancer grades. Our research, conducted at the Baqiyat Elah Hospital in Tehran in 2021, involved collecting blood and serum samples from BC patients. These samples underwent BRCA1 gene expression analysis and CA15-3 tumor marker assessment. Using the AJCC grading system, we categorized BC patients into various grades. Our findings revealed that BRCA1 gene expression was present in 28.57% of patients, while 71.43% showed negative expression. Both BRCA1 expression and CA15-3 levels significantly increased with advanced cancer stages (*P* < 0.001). These results suggest the potential utility of BRCA1 gene expression and CA15-3 tumor marker assessment in BC prognosis and management, particularly concerning staging and disease progression. This study provides valuable insights into the biology of BC and the development of prognostic markers for improved patient outcomes.

## 1. Introduction

Cancer, caused by DNA sequence changes, accounts for one in eight deaths worldwide [1]. BC, a major type of malignant neoplasm, primarily affects the inner layers of mammary glands or ducts and has a high incidence in Asia [2]. Globally, it is the most common cancer among women, with 1.5 million new cases and 500,000 deaths annually [3]. Incidence rates are highest in North America and Western Europe and lowest in Asia and Africa [4, 5]. In Iran, BC is the most prevalent cancer among women [6, 7]. Staging, crucial for treatment, typically uses the TNM system, established by the American Joint Committee on Cancer in 1959 [8]. According to the number of affected lymph nodes, it is classified as stage I without lymph node involvement, stage II when one to three lymph nodes are affected, and stage III when more than four lymph nodes are involved [9]. BC includes several genetic and environmental factors [10]. Compared to racial and genetic factors, environmental factors are more easily controlled. Other risk factors include diet, especially high in fat, smoking, family history of BC, depression, and stress [11]. Genetic predisposition due to mutations in autosomal dominant genes accounts for 5 to 10% of all breast malignancies. The genetic diversity involved in breast tumor development includes two broad types: loss of function mutations in tumor suppressor genes, leading to uncontrolled cell growth, disrupted cell cycle checkpoints, and DNA repair failure; and gain of function mutations in proto-oncogenes. Women with an inherited functional mutation face a 70% risk of developing BC before the age of 70 [12]. BRCA1, a tumor suppressor gene, has three main domains, including a ubiquitin ligase domain (RING domain) at the N-terminal [13]. The search for new prognostic markers to improve survival prediction is ongoing, with increasing attention on the impact of tumor markers. Cancer antigen (CA15-3), part of the mucin-1 (MUC-1) glycoprotein family, is overexpressed in cancers and is recognized as a useful tumor marker due to its altered glycosylation [14]. Genetic risk identification through BRCA1 analysis enables tailored treatment decisions, while monitoring disease dynamics with CA 15-3 facilitates timely intervention, ensuring effective care. This holistic approach empowers patients with early detection insights, optimizing BC management and improving outcomes [15]. As a result, this study aimed to compare BRCA1 gene expression and CA15-3 tumor marker levels across different BC stages, focusing on their correlation with cancer stage and potential utility in prognosis and disease management. The research sought to provide insights into the role of these markers in staging and disease progression.

## 2. Materials and Methods

This study was conducted on women with BC referred to the cancer research center of Shahid Beheshti Medical University in Tehran in 2021.

### 2.1. Sampling

First, an experienced and skilled nurse used a sterile syringe to collect 5 cc blood samples and 5 cc serum samples from women with BC, whose diagnoses were confirmed by a specialist doctor. The serum samples were then transferred to the immunology laboratory for the assessment of the CA15-3 tumor marker, while the blood samples were simultaneously transported to the molecular laboratory for BRCA1 gene analysis.

### 2.2. Staging

The well-known method of the American Joint Committee on Cancer (AJCC) was used to determine the grade of BC in patients.

### 2.3. RNA Extraction

Based on the protocol of the MACHEREY NAGEL kit, RNA was extracted from all collected whole blood samples. The quality of the extracted RNA was then assessed using electrophoresis in 1% agarose gel.

### 2.4. cDNA Synthesis

To eliminate DNA contamination, DNase I treatment was performed using a DNase reagent (Sinnaclone, Iran) according to the manufacturer's instructions, ensuring the prevention of genomic DNA contamination and minimizing nonspecific binding. For cDNA synthesis, the TOPscript™ cDNA Synthesis Kit (Enzynomics, Republic of Korea) was employed. Each reaction utilized 1 *μ*g of RNA sample. The treated RNA was then subjected to reverse transcription using random hexamer primers and the cDNA synthesis kit, following the manufacturer's instructions.

### 2.5. Primer Design

In this study, BRCA1 was the target gene and GAPDH was used as the housekeeping gene. Primers were designed using NCBI, HGNC, and GeneCards data, followed by comparison and preparation in Clustal Omega, Gene Runner, and BLAST. The primer sequences were as follows.

### 2.6. Quantitative Real-Time PCR (RT-qPCR)

In the real-time PCR setup, 1 *μ*l of cDNA was combined with SYBR green qPCR master mix 2X (Yekta-Tajhiz-Azma, Iran). The internal control gene used was GAPDH. Primers for amplifying the target genes were designed using Oligo 7 software, Protocol Online, and OligoAnalyzer Tool. A BLAST analysis was conducted to ensure primer specificity. The primer sequences can be found in Table 1. The cycling conditions for 35 cycles of RT-PCR are detailed in Table 2.

### 2.7. Measurement of Tumor Marker CA15-3

CA15-3 serum levels were quantified using an automated electrochemiluminescence immunoassay system (ROCHE E170; Roche, Germany). Levels above 25 *μ*g/L were considered high, as per established cutoff points for the CA15-3 tumor marker.

### 2.8. Statistical Analysis

The Ct values obtained from RT-qPCR were employed in the 2^−ΔΔCT^ method to assess relative fold expression changes. Descriptive statistics included the mean and standard deviation for normally distributed data and median with interquartile range (IQR) for non-normal distributions. Chi-square tests were used for categorical comparisons of BRCA1 gene expression across variables and cancer stages. Mann–Whitney and Kruskal-Wallis tests were applied to compare CA15-3 tumor marker levels due to their suitability for nonparametric data. Spearman's partial correlation examined associations between CA15-3 and BRCA1 expression across different BC stages, controlling for relevant factors. Data were analyzed using Stata software (version 14), with significance set at *P* < 0.05 for robust statistical evaluation.

## 3. Results

### 3.1. Brief Explanations

The study included 70 BC patients with an average age of 56.4 years. Positive BRCA1 expression was observed in 28.57% of patients and was associated with advanced cancer stages. CA15-3 levels also showed a significant increase with advanced stages. Furthermore, a strong positive correlation was found between CA15-3 levels and BRCA1 expression in advanced cancer stages. Fisher's exact test indicated a nonsignificant trend for BRCA1 expression and a history of ovarian cancer (*P*=0.12). These findings provide valuable insights into the relationships between patient demographics and biomarkers analyzed in the study.

### 3.2. Descriptive Statistics

**Figure 1:**
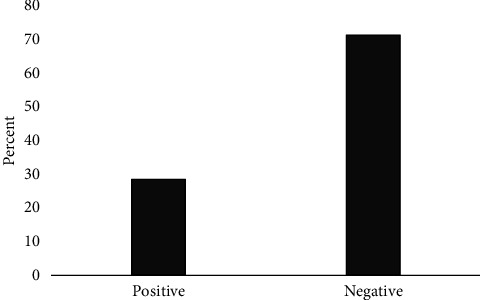
BRCA1 gene expression frequency in BC patients.

This study involved 70 BC patients with an average age of 56.4 ± 12.57 years, with 77.14% aged over 50. Notably, 86.22% had a history of BC, 18.57% of ovarian cancer, and 35.71% reported a family history of cancer. The distribution of cancer stages ranged from stage 1 (54.29%) to stage 4 (2.86%). Positive BRCA1 gene expression was observed in 28.57% of patients (20 individuals), while 71.43% showed negative expression (Figure 1).

As shown in Table 3, the mean BRCA1 gene expression was 35.85 ± 4.35 for patients under 50 years of age and 35.00 ± 3.94 for those 50 years and older, with no statistically significant difference observed (*P*=0.710). Patients with a history of BC exhibited a significantly higher mean BRCA1 gene expression of 39.11 ± 4.29 compared to those without such a history (34.04 ± 3.14), with a *P* value less than 0.001. Analysis of ovarian cancer history revealed no statistically significant difference in BRCA1 gene expression between patients with a positive history (36.24 ± 4.02) and those without (34.96 ± 4.02, *P*=0.120). However, patients with a positive family history of cancer showed a significantly higher mean BRCA1 gene expression of 37.29 ± 4.43 compared to those without (34.03 ± 3.28, *P*=0.001). Regarding BC stage, patients with stage 1 had a mean BRCA1 gene expression of 33.47 ± 2.49, while those with stages 2, 3, and 4 exhibited mean expressions of 36.57 ± 4.54, 39.50 ± 4.90, and 38.55 ± 0.01, respectively. The differences in BRCA1 gene expression across these stages were statistically significant (*P* < 0.001), indicating a trend of increased BRCA1 gene expression with higher stages of BC (Table 3).

The median CA15-3 level was 32.85 *μ*g/L (IQR: 14.85) for patients under 50 years of age and 33.95 *μ*g/L (IQR: 57.30) for those 50 years and older, with no statistically significant difference observed (*P*=0.820). Patients with a history of BC had a significantly higher median CA15-3 level of 190.2 *μ*g/L (IQR: 700) compared to those without such a history (28.55 *μ*g/L, IQR: 13.5), with a *P* value less than 0.001. Similarly, patients with a positive history of ovarian cancer showed a median CA15-3 level of 78.8 *μ*g/L (IQR: 172), significantly higher than those without such a history (29 *μ*g/L, IQR: 17.5, *P* < 0.001). Patients with a positive family history of cancer had a median CA15-3 level of 70 *μ*g/L (IQR: 172.2), while those without a family history had a median of 26.4 *μ*g/L (IQR: 14.2), with a significant difference observed (*P* < 0.001). Additionally, patients with positive BRCA1 gene expression had a median CA15-3 level of 539.3 *μ*g/L (IQR: 105.9, *P* < 0.001). In terms of BC stage, patients with stage 1 had a median CA15-3 level of 26.5 *μ*g/L (IQR: 15.2), while those with stages 2, 3, and 4 had median levels of 38.9 *μ*g/L (IQR: 37.05), 584.5 *μ*g/L (IQR: 548), and 977 *μ*g/L (IQR: 46), respectively. The differences in CA15-3 levels across these stages were statistically significant (*P* < 0.001), indicating an association between higher stages of BC and increased CA15-3 levels (Table 4).

The study conducted Spearman's partial correlation analysis to explore the relationship between the CA15-3 tumor marker and BRCA1 gene expression across different stages of BC, while controlling for various factors (Table 5). For patients with stage 1 BC, the partial correlation coefficient (*r*) was 0.222 (*P*=0.185) when adjusted for age alone, and 0.248 (*P*=0.150) when further adjusted for age, breast and ovarian cancer history, and family history of cancer. These findings suggest that the correlation between CA15-3 levels and BRCA1 gene expression was not statistically significant in stage 1 BC patients. Similarly, for patients with stage 2 BC, the partial correlation coefficient (*r*) was 0.278 (*P*=0.197) when adjusted for age alone and 0.138 (*P*=0.549) when further adjusted for the additional factors. These results indicate that like stage 1, the correlation between CA15-3 and BRCA1 gene expression was not statistically significant in stage 2 BC patients.

In contrast, a different trend was observed in patients with stages 3 and 4 BC. When adjusted for age, the partial correlation coefficient (*r*) was 0.463 (*P*=0.295). Upon further adjustment for breast and ovarian cancer history and family history of cancer, the partial correlation coefficient significantly increased to 0.959 (*P*=0.009). These findings highlight a strong and statistically significant positive correlation between CA15-3 levels and BRCA1 gene expression in advanced stages of breast cancer (stage 3 and 4), even after accounting for potential confounding factors (Table 5).

## 4. Discussion

Between 5% and 10% of BC have an inherited component and are often associated with ovarian cancers. The risk is particularly associated with high-penetrance susceptibility genes, including BRCA1 located on chromosome 17q21 [16, 17]. Women with mutations in BRCA1 have a 57% to 65% chance of developing BC in their lifetime [12]. Germ-line mutations in the BRCA1 gene contribute to approximately 25% of familial BC, while somatic inactivation of BRCA1 is found in up to 5% of sporadic BCs [18–20]. The role of tumor markers in diagnostics has become increasingly important. Carcinoembryonic antigen 15-3 (CA15-3), a member of the mucin-1 (MUC-1) glycoprotein family, is recognized as a valuable tumor marker. It is typically overexpressed in cancers, a characteristic that is accentuated by altered glycosylation patterns [21, 22]. In our study, 28.57% of patients exhibited positive BRCA1 gene expression, contrasting with 71.43% who tested negative. The mean and median CA15-3 tumor marker levels were 112.53 ± 231.88 and 33.95 (IQR = 42.2), respectively. Comparative studies revealed varying BRCA1 expression rates: Brianese et al. reported 20.6%, and Goldzik et al. found 24.2%. Additionally, Li et al. documented a CA15-3 expression rate of 54.95%, while Zhao et al. reported a lower rate of 5.62% in BC patients [23–26]. Family history is a well-known factor significantly impacting BC risk, with an odds ratio of 1.71 [27]. Another study involving a large patient cohort indicated that women with two or more relatives with a history of BC have a 2.5-fold increased risk of developing the disease [28]. These findings underscore the importance of family history in BC screening and prevention strategies.

Our study identified a significant correlation between BRCA1 gene expression and CA15-3 tumor marker levels among individuals with a history of breast and ovarian cancer. We also observed lower expression levels of these markers in individuals under 50 years compared to those aged 50 or older. Moscatello et al. emphasized the critical role of examining the BRCA1 gene in individuals with a prior history of BC. Jiang et al., in a study involving 3,217 BC cases, reported a positive correlation between BRCA1 and BRCA2 gene expressions and a family history of the disease. Udonkong et al. highlighted the significance of BRCA1 gene expression in BC patients over 50, while Nam et al. underscored the importance of CA15-3 and CEA tumor markers in BC patients with a history of the disease [29–32].

Women with inherited mutations in BRCA1 or BRCA2 face an elevated risk of developing breast and ovarian cancers [17]. Females carrying BRCA1 mutations have a 57–65% likelihood of developing BC during their lifetime [12]. Germ-line mutations in the BRCA1 gene account for about 25% of familial BCs, while somatic inactivation of BRCA1 is found in up to 5% of sporadic cases [18, 19]. Numerous studies have highlighted the association between BRCA1, BRCA2 gene expression, and BC [33–35].

Cancer antigen 15-3 (CA15-3), a member of the mucin glycoprotein family (MUC1), is widely used as a tumor marker in breast cancer patient management. Elevated CA15-3 levels can also be detected in early stages but are more commonly associated with metastatic BC [36]. High CA15-3 levels are also found in various carcinomas and benign diseases [37]. However, there is limited research comparing the expression of these genes across different stages of BC. In this meta-analysis study, we aimed to investigate not only the expression levels of BRCA1 and CA15-3 genes but also their variations across different stages and grades of BC.

In our study, the frequency of BRCA1 gene expression significantly increased with advancing disease stages, reaching 100% in stage +4. Similarly, CA15-3 levels exhibited a significant rise with increasing disease stages, with the highest mean observed in stage +4 (977 *μ*g/L). These findings are consistent with Saif et al.'s study on 50 BC samples, which also reported an increase in BRCA1 expression with higher stages. Song et al.'s study involving 332 samples further supported our results, highlighting a correlation between BRCA1 expression and advanced disease stages (*P* < 0.001) [38, 39].

In this study, CA15-3 gene expression was assessed across different BC stages, revealing the highest mean in stage +3, followed by +2, and the lowest in +1. Importantly, mean CA15-3 levels increased significantly with advancing stages, consistent with findings from Li et al.'s study where CA15-3 expression rates were separately reported as 6.9% for stage 1, 8.8% for stage 2, and 18% for stage 3 [25]. Araz et al.'s investigation similarly documented varying CA15-3 expression rates in BC stages 1 (18.73%), 2 (16.02%), and 3 (19.16%) [40].

## 5. Conclusion

According to the findings of this study, assessing the levels of BRCA1 gene expression and CA15-3 tumor marker in BC patients, particularly those with a family history of breast or ovarian cancer, is crucial. The study highlights that these markers increase with higher cancer stages, underscoring their importance in patient identification and prognosis. Further research in this area is warranted to deepen our understanding and clinical applications.

## Figures and Tables

**Table 1:** The primer sequences.

Primers	Sequences
BRCA1 forward	5′-TTGTCAATCCTAGCCTTCCAAGAG-3′
BRCA1 reverse	5′-GCGCTTTGAAACCTTGAATGTAT-3′
GAPDH forward	5′-CGACCACTTTGTCAAGCTCA-3′
GAPDH reverse	5′-AGGGGTCTACATGGCAACTG-3′

**Table 2:** PCR temperature table.

Function	Temperature (°C)	Time	No. of cycles
Initial temperature	94	4 min	1
RT-PCR			
Denaturation	94	30 s	35
Annealing	60	30 s	
Extension	72	20–30 s	
Final expiration temperature	72	4 min	1

**Table 3:** Comparison of BRCA1 gene expression by different variables in the studied patients.

Different variables	Mean ± SD	Positive (*N* = 20)	Negative (*N* = 50)	*P* value^*∗*^
		Percent (%)	Percent (%)	
Age categories				
Less than 50 years	35.85 ± 4.35	4 (25)	12 (75)	0.710
Equal to/more than 50 years	35.00 ± 3.94	16 (29.63)	38 (70.37)	
Breast cancer history				
Yes	39.11 ± 4.29	13 (81.25)	3 (18.75)	<0.001
No	34.04 ± 3.14	7 (12.96)	47 (87.04)	
Ovarian cancer history				
Yes	36.24 ± 4.02	6 (46.15)	7 (53.85)	0.120
No	34.96 ± 4.02	14 (12.56)	43 (75.44)	
Family history of cancer				
Yes	37.29 ± 4.43	13 (52)	12 (48)	0.001
No	34.03 ± 3.28	7 (15.56)	38 (84.44)	
Stage of breast cancer				
1+ (*N* = 38)	33.47 ± 2.49	0 (0.00)	38 (100)	<0.001
2+ (*N* = 24)	36.57 ± 4.54	13 (54.17)	11 (45.83)	
3+ (*N* = 6)	39.50 ± 4.90	5 (83.33)	1 (16.67)	
4+ (*N* = 2)	38.55 ± 0.01	2 (100)	0 (0.00)	

^*∗*^Based on the chi-square test.

**Table 4:** Comparison of CA15-3 tumor marker distribution (*μ*g/L) by different variables in the studied patients.

Variables	Median (IQR)	Min	Max	*P* value^*∗*^
Age categories				
Less than 50 years (*N* = 16)	32.85 (14.85)	16	369	0.820
Equal to/more than 50 years (*N* = 54)	33.95 (57.30)	5.9	1000	
Breast cancer history				
Yes (*N* = 16)	190.2 (700)	15.16	1000	<0.001
No (*N* = 54)	28.55 (13.5)	5.9	13.97	
Ovarian cancer history				
Yes (*N* = 13)	78.8 (172)	23.2	1000	<0.001
No (*N* = 57)	29 (17.5)	5.9	1000	
Family history of cancer				
Yes (*N* = 25)	70 (172.2)	15.1	1000	<0.001
No (*N* = 45)	26.4 (14.2)	5.9	800	
Expression of BRCA1				
Yes (*N* = 20)	539.3 (105.9)	34.4	1000	<0.001
Stage of breast cancer				
1+ (*N* = 38)	26.5 (15.2)	5.9	137.9	<0.001^*∗∗*^
2+ (*N* = 24)	38.9 (37.05)	15.16	212	
3+ (*N* = 6)	584.5 (548)	34.3	1000	
4+ (*N* = 2)	977 (46)	954	1000	

^*∗*^Based on the Mann–Whitney test; ^*∗∗*^based on the Kruskal–Wallis test.

**Table 5:** Spearman's partial correlation between CA15-3 tumor marker and BRCA1 gene expression by cancer stage in the studied patients.

Stage of breast cancer	*r* ^*∗*^	*P* value	*r* ^*∗∗*^	*P* value
1+ (*N* = 38)	0.222	0.185	0.248	0.150
2+ (*N* = 24)	0.278	0.197	0.138	0.549
3+ and 4+ (*N* = 8)	0.463	0.295	0.959	0.009

^*∗*^Adjusted for age, adjusted for age, breast and ovarian cancer history, and family history of cancer. *r*^*∗*^ indicates a partial correlation coefficient significant at a specific level (e.g., *p* < 0.05), marked by the asterisk. *r*^*∗∗*^ represents a partial correlation coefficient significant at a stricter level (e.g., *p* < 0.01), marked by the double asterisk (^*∗∗*^), indicating higher significance than *r*^*∗*^.

## Data Availability

Ethical guidelines prevent the release of specific metadata related to clinical data involving human subjects. Consequently, we are unable to provide access to a summary of the patients' data.
